# Diversifying the Utilization of Maize at Household Level in Zambia: Quality and Consumer Preferences of Maize-Based Snacks

**DOI:** 10.3390/foods10040750

**Published:** 2021-04-01

**Authors:** Emmanuel Oladeji Alamu, Bukola Olaniyan, Busie Maziya-Dixon

**Affiliations:** 1 International Institute of Tropical Agriculture (IITA), Southern Africa Research and Administration Hub (SARAH) Campus, P.O. Box 310142, Chelstone, Lusaka 10101, Zambia; 2 International Institute of Tropical Agriculture (IITA), PMB 5230, Ibadan 20001, Nigeria; oluwatoyinolaniyan086@gmail.com (B.O.); B.Maziya-Dixon@cgiar.org (B.M.-D.)

**Keywords:** maize snacks, nutritional characterization, consumer preferences

## Abstract

This study evaluated the nutritional, antinutritional properties, and consumer preferences of five maize-based snacks at the household level. The physical, nutritional, and antinutritional properties were analyzed with standard laboratory methods, while a structured questionnaire was used for the data collection on consumer preferences of the maize products. There were significant (*p* < 0.05) differences in the proximate parameters of the maize snack samples. Antinutritional properties among maize snacks all fell within the permissible range. Respondents from all districts showed no significant (*p* > 0.05) differences in maize chin-chin variants’ and maize finger variants’ except for Serenje and Mkushi districts where maize chin-chin and maize finger showed significant (*p* < 0.05) differences in their sensory ratings. However, across districts, the most rated maize finger variant was the spiced 100% maize finger. In conclusion, maize-based snacks enriched with soybean flour have proven nutritious with a reasonable acceptability level.

## 1. Introduction

Maize serves as the nutritional backbone in central, southern, and eastern Zambia [1]. It is the main staple, providing 52% of the local population’s daily calorie intake [2]. Maize is regarded as an economic and political crop in Zambia due to enabling maize production across the country [3]. Approximately 80% of Zambian smallholder households and 20% of commercial farmers grow maize. Many produce other crops such as cassava, grown mainly as a food security crop and soybean as a cash crop. However, there has been substantial financial input from the Government of Zambia in maize production. In 2006, Zambia ranked 13th among 51 countries prominent for maize production in Africa, with a total of 0.865 million tons, and this increased to 3.607 million tons by 2016 [4]. According to FAOSTAT [5], Zambia ranked eighth among the top ten maize producing countries in sub-Saharan Africa [6]. Despite government interventions in the maize sector, there were continuous fluctuations in productivity due to prolonged dry seasons and short rainy seasons [4]. 

In the bid to increase maize yield in Zambia, improved varieties prominent for grain yield, dry matter, and water usage efficiency in areas of low and erratic rainfall were introduced to farmers [1,7]. This was made possible through the collaborative research of HarvestPlus, International Maize and Wheat Improvement Center (CIMMYT), IITA, the Zambia Agriculture Research Institute (ZARI), and other partners who developed maize varieties with qualities such as high yield, disease resistance, consumer acceptability, and high provitamin A carotenoid content [1,8]. The outcome made a positive impact on the food security status of farm households [1]. However, maize’s household utilization has been limited to traditional products such as “Nshima” and various local beverages that are not nutritious enough for household consumption [9]. The National food price data reveals that, as the overall cost of food is reducing, foods high in nutrients are more expensive than staple foods [10]. Hidden hunger and undernutrition have been public health issues in Zambia among children and adults alike [11,12]. A recent study suggested that close to 60% of inpatients at a teaching hospital were at nutritional risk, as pre-admission nutritional status was an associated factor [13].

Furthermore, to combat undernutrition/hidden hunger, most especially Fe and Zn deficiencies, there is a need for swift action such as food-to-food fortification. Regular staples consumed without adequate protein have contributed to low dietary diversity, so utilizing the available and preferred staple to create diversity in nutrient-dense foods at an affordable cost is imperative. However, maize is notorious for phytic acid, which binds with proteins and essential minerals such as Fe and Zn [14]. Nevertheless, different processing methods usually reduce it to safe levels for humans; furthermore, new maize germplasms are specifically bred to be low in phytic acid [15]. Thus, product development using maize and legumes (e.g., soybean) can diversify maize’s household utilization.

Legumes are one of the world’s most valuable food supply sources, particularly in developing countries where food, energy, and nutrients are of utmost concern [16]. They have been recognized as an essential protein source with maximum advantageous bioactive compounds such as minerals and fat-soluble vitamins, particularly soybean [17]. They are well-laden with sulfur-containing amino acids; the most prominent are Lysine and Tryptophan, mostly not found in cereals. They also possess digestible protein [17,18]. Soybean is unique due to its ability to adjust to many soils and climates and its nitrogen fixative ability [19]. This ability qualifies it as an ideal rotational crop for nitrogen fixation for maize crops, especially in a country such as Zambia.

Snacks are convenience foods that have been around for a long time, but demand increased with urbanization and population [19]. Adebowale and Komolafe [20] reported varieties of snacks and dishes produced from maize familiar to the Nigerian populace. Most snacks are made from cereals, but several findings have exposed their low nutrient concentration [21], which can be made up for by fortification or blending with legumes such as soybean [22], groundnut [16], African yam bean [17], cowpea [18], and pigeon pea [23]; also, defatted coconut has been used in the fortification of maize deep-fried snacks [20]. 

Zambia produces maize on a relatively large scale, so most of the time, the country exports the surplus to neighboring countries. Diversifying maize for snack production at the household level will enhance the maize value chain and improve the nation’s nutritional status. Thus, the study aimed at evaluating the physical, nutritional, and antinutritional properties and consumer preferences of five maize-based snacks produced from maize and soybean. 

## 2. Materials and Methods

### 2.1. Materials

Maize grains, soybean grains, salt, sugar, margarine, baking powder, and vegetable oil were purchased at local Zambian markets.

### 2.2. Processing Maize Grain to Flour

Maize flour was produced as described by Adeola et al. [24]. The maize grains were dried, sorted, and cleaned to remove stones, dirt, and infested grains. The cleaned maize was then milled using a laboratory hammer mill and passed through a 100-micrometer mesh sieve.

### 2.3. Processing Soybean to Flour

The soybean flour was prepared using the methods described by Alamu et al. [25]. The grains were cleaned and sorted to eliminate stones and other undesirable materials. The cleaned soybean seeds were roasted slightly at a temperature of about 120 °C for 5 min until the seed coat was loose and easily removed by hand. The roasted seeds were then coarse-milled and winnowed to remove the seed coat. The decorticated soybean was finely milled to 0.5 mm particle size using a laboratory mill (Perten, Hägersten, Sweden) to obtain fine flour. The flour was packaged and appropriately stored before use.

### 2.4. Maize-Based Products

Five maize-based products: Plain maize finger, spiced maize finger, spiced fortified maize finger, plain maize chin-chin, and fortified maize chin-chin were prepared using 100% high-quality maize flour (HQMF), blended HQMF, and soybean flour (80:20). Plain maize finger and plain maize chin-chin prepared from HQMF were the control in this experimental setup. Table 1 gives a summary of the ingredients weighed in grams for all products.

#### 2.4.1. Processing Maize Finger (Kokoro)

A total of 250 g of HQMF was stirred into 625 mL of hot water (95 °C) to form a thick porridge through the maize starch’s gelatinization. The porridge was added to the remaining portion of salted 250 g of maize flour to form a sticky dough. This was mixed thoroughly using an electric mixer and then left to cool to room temperature. A small portion of the dough was scooped, smoothened with the palm, and rolled either on the palm or on a smooth surface to form a firm, long-shaped dough. Then it was deep-fried in 2 L of “Ole oil” (Sunflower oil) at the temperature range of 170 to 175 °C for an average of 4.5 min. The color changed from off-white to brown depicting a Maillard reaction, and a long-shaped, crunchy snack was produced. 

#### 2.4.2. Processing Maize Finger Fortified with Soy Flour

The processing method for fortified maize finger is the same as plain maize finger. The only difference is the incorporation of 125 g of soy flour added to the remaining portion of salted 250 g of HQMF. Further, the frying time was extended by 30 s, a little longer than for plain maize finger. It took an average of 5 min for the Maillard reaction to be completed. Table 1 explains all the ingredients and quantities added in grams. 

#### 2.4.3. Processing Maize Chin-Chin

Five hundred grams of HQMF was mixed with all the dry ingredients in a bowl; 100 g sugar and 10 g baking powder. Forty grams of margarine was added and mixed thoroughly. Two medium-sized eggs with an average weight of 62.2 g and 250 mL of room temperature water were added to form a dough, kneaded to ensure dough uniformity. 

The dough was spread on a smooth, clean surface and sprinkled with HQMF to prevent stickiness. A stainless-steel knife was used to cut the dough into small rectangular shapes, which were deep-fried in 2 L of “Ole oil” (Sunflower oil) within a range of 170 to 175 °C as frying progressed. It took an average of 5 min for frying to be completed, and the product was strained and cooled on a clean flat surface; the result was a sweet, crunchy snack.

#### 2.4.4. Processing Maize-Soy Chin-Chin

The processing is the same as for the plain chin-chin. The fortifier, 125 g of High Quality Soy flour (HQSF), was added to the maize flour when all other dry ingredients were added. It took an average frying time of 5 min for the Maillard reaction to be completed when the frying sample became brown. 

#### 2.4.5. Processing Spicy Maize Finger (Kokoro)

The processing follows the same as that for maize finger and maize-soy finger. The significant difference is the addition of two medium-sized onions with an average weight of 63.8 g each as a spice.

### 2.5. Determination of Nutritional and Physico-Chemical Properties of Maize Snacks

The maize finger, maize-soy finger, maize chin-chin, and maize-soy chin-chin were analyzed for moisture, protein, fat, ash, total reducing sugars, total starch, digestible starch, non-digestible starch, amylose, phytate, tannins, pH, bulk density, and color parameters in duplicate

1. Moisture content determination: The pulverized samples were used to determine the moisture content using the method reported by Alamu et al. [25].
2. Ash content determination: The method of AOAC [26] as reported by Alamu et al. [27].
3. Protein content determination: The Kjeldahl method was used to determine the protein content by multiplying the nitrogen value with a conversion factor of 6.25, as described by Alamu et al. [25].
4. Crude fat content determination: The Soxhlet extraction method was used as described by Alamu et al. [27].
5. Digestible starch and total reducing sugar content determination: Digestible starch and total reducing sugar were determined using the Dubois method [28], as reported by Alamu et al. [25].
6. Amylose content determination: The adapted method Williams et al. [29] was used to determine amylose content described by Alamu et al. [25].
7. Carbohydrate content: This was derived by calculating the difference, %CHO = 100—(sum of the percentages of moisture, ash, fat, protein, and crude fiber)

### 2.6. Determination of Antinutritional Properties of Maize Snacks

1. Phytic acid content: Phytate was determined by the extraction and precipitation of phytic acid according to Wheeler and Ferrel’s method [30] as described by Okukpe & Adeloye [31].
2. Tannin content determination: Tannins were determined by the method described by da Silva Lins et al. [32].

### 2.7. Determination of Functional Properties of Maize Snacks

1. pH determination: This was done using 10 g of pulverized maize finger and maize chin-chin dispersed in 20 mL of deionized water to detect the suspension’s pH using a table-top pH meter [33].
2. Bulk density determination: Bulk density was determined using the method recommended by AOAC [26]. The sample (7 g) was placed into a 50 mL graduated measuring cylinder and then tapped gently against the palm until a constant volume was obtained.
3. Color parameters: Color measurements were performed on pulverized samples using a color meter. The color of products was expressed as the average of three L*, a*, and b* readings, where L* stands for brightness, a* redness, a* greenness, b* yellowness, and b* blueness. A white calibration plate was used to standardize the equipment before color measurements [34].

#### 2.8. Sensory Evaluation and Consumer Preferences of Maize Snacks

The maize finger’ quality attributes made from 100% corn flour, spiced 100% maize finger, spiced 80:20% maize-soy finger, 100% maize chin-chin, and 80:20% maize-soy chin-chin were assessed by a 30-member sensory panel. The panelists were well trained on the desired descriptors for all maize products, and all precautions were adhered to strictly before the sensory session. Forms were administered, and panelists were asked to score the samples using the 9-point hedonic scale for taste, color, crispiness, flavor, and overall acceptability. The scores were ranked and subjected to statistical analysis.

The investigation was conducted in each major maize-growing district (Monze, Katete, Serenje, and Mkushi). The areas were selected based on levels of consumption and accessibility. Four hundred and thirteen respondents (413) were available for the survey, with 104 respondents in Monze, 109 in Katete, 108 in Serenje, and 92 in Mkushi. They were all randomly selected. The data was collected using a well-designed questionnaire administered to each respondent by well-trained enumerators. They were adequately informed about the study, and an agreement was reached to obtain their consent. The maize-based products were well coded to avoid a mix-up and presented to the participants randomly according to the method described. The sensory attributes chosen were aroma, appearance, taste, texture, and overall acceptability. The attributes of each product were rated by participants on a 5-point hedonic scale to measure the degree of likeness using qualitative judgments that correspond to 1 = dislike very much, 2 = dislike a little, 3 = neither like nor dislike, 4 = like a little, 5 = like very much. The sensory testing order was such that product appearance was rated first, then aroma, and finally taste and texture. Clean, potable water was supplied to respondents for necessary rinsing of the mouth between one product and another to be precise with each product’s sensorial attributes [35]. 

### 2.9. Statistical Analysis

The data generated on the proximate, functional, and antinutritional properties were statistically analyzed using IBM SPSS statistical software (Version 21). The data about preference and willingness to consume were subjected to Analysis of Variance (ANOVA) at a 95% level of significance. The differences between means were considered significant at *p* < 0.05 using the Duncan multiple range test.

## 3. Results and Discussion

### 3.1. Nutritional Properties of Deep-Fried Maize-Based Snacks

Table 2 shows the results for nutritional and antinutritional properties of deep-fried maize-based snacks. All maize snacks’ moisture content was minimal and did not harm the products’ quality attributes.

The addition of soy flour and eggs caused an increase of about 50% ash content in the products. It implies that Fe, Zn, and other minerals contents will be markedly higher in the soy-fortified maize products than 100% maize products. All products seem to be high in fat content, mainly due to deep frying. This could have a negative effect on the storability of the products due to unsaturated fatty acid exposure to warm or hot air known as oxidative rancidity [16]. The same trend was observed as reported by [36] that some snacks’ high-fat content resulted from the processing techniques that involved the addition of cooking oils and/or deep-frying. 

The protein values obtained for the snacks are significantly (*p* < 0.05) different. The addition of legumes to cereals has been scientifically established to improve its protein quality [37,38,39]. Soy flour is rich in sulfur-containing amino acids such as lysine, which is deficient in maize or eroded. So the addition of soy flour or other legumes enhances maize snacks’ nutrient content. 

Furthermore, all products’ amylose content is highly significant at *p* < 0.001. Research has expounded on the importance of amylose in diets. It positively correlates with resistant starch by slowing down glucose release into the bloodstream, thereby benefiting those managing obesity or hyperinsulinemia [9]. The sugar content across all products has a high significance at *p* < 0.001, with 100% maize finger (kokoro) having the lowest value of 1.88% while 100% maize chin-chin has the highest value of 3.45%. The trend observed is that products fortified with soy flour have an elevated sugar level than their unfortified counterparts. A similar pattern was reported by [24] where the sugar content of kokoro increased as AYF (African yam bean flour) substitution increased. Another possibility is that 100% maize chin-chin records a high sugar content due to sucrose as part of the recipe.

The product that has the highest starch content is the 100% maize finger (62.73%), while 20% soy-maize finger and spiced 20% soy-maize finger have the lowest values of 42.14% and 48.71%, respectively. Maize is predominantly starch accounting for 60–75% of the kernel, and so the starch content is appreciably reduced when substituted with legumes such as soybean. This same trend was reported by [24] when 100% of maize kokoro had the highest starch content than other products substituted with AYF. The total carbohydrate across the products shows no significant (*p* > 0.05) difference except 100% maize finger and its spiced variant. This same observation was reported by [16] and [25], who substituted maize snack (kokoro) with partially defatted groundnut paste and cowpea flour, respectively. The high total carbohydrate values observed for 100% maize finger and its spiced variant could be from starch hydrolysis. This suggests that the 100% maize products (maize finger and chin-chin) record the highest total digestible carbohydrates. This can be explained by their starch structure with high amylopectin, which results in a high degree of branching, a disrupted granular structure of starch, thus increasing its susceptibility to the attack by enzymes and in-vitro digestibility [40]. Although the consumption of 100% maize products may rapidly boost energy levels, especially when an instant burst of energy is required, such as during an endurance sporting event, rapidly digestible carbohydrate is the best choice to make. Therefore, glucose tablets and sports drinks are so popular [40].

Nevertheless, there may also be a tendency to raise blood glucose levels, resulting in hyperglycemia in some individuals, particularly those with impaired glucose tolerance [41]. Alternatively, maize products fortified with soy flour and the spiced 100% maize finger have high values for total non-digestible carbohydrates, with 20% soy-maize chin-chin having the highest value of 24.15%. This qualifies the product to fit into the carbohydrates group known as resistance starch, generally referred to as dietary fiber. This non-digestible carbohydrate is absorbed in the small intestine. It ferments, to some extent, producing short-chain fatty acids with advantages such as improvements in glycemic control, bowel health, and cardiovascular disease prevention [42]. The naturally occurring ones such as verbascose and low molecular weight fructans are found in legumes and onions [40]. This justifies why 20% soy-maize chin-chin has the highest value of 24.15% and the spiced 100% maize finger 18.75%, the second-highest value.

The 20% soy-maize chin-chin has the highest energy value even though there is no significant (*p* > 0.05) energy difference among the products. This agrees with [39], who reported an increase in the energy value with the increasing proportion of pigeon-pea protein concentration in maize flour kokoro. Furthermore, protein and fat are usually associated with high calorific values in food. The relatively high-fat content across the products resulting from deep-frying contributed immensely to the energy value. 

The antinutritional properties across the products show no significant (*p* > 0.05) differences for tannin and phytate. The lowest value for tannin was observed in 100% maize finger with a value of 1.13 mg/g, and the highest value observed in 20% soy-maize chin-chin. The products containing soy were observed to have the most tannin concentration, while the highest values for phytate were seen in the spiced 20% soy-maize with a value of 2.33% and the lowest value of 1.23% observed in 100% maize chin-chin. Tannins are known to be notorious for reducing the bioavailability of proteins in humans and animals and notable for their antioxidative and anti-inflammatory characteristics [43]. Phytate also forms complexes with essential minerals and protein in foods, making them unavailable for absorption and deactivating digestive enzymes [19]. Both antinutrients are significantly found in cereals and legumes but usually reduced when subjected to processing such as dehulling, cooking, frying, malting, fermentation, and oven-drying, among others [15]. Hence, the values obtained across all products from Table 2 are considered safe enough for human consumption.3.2. Physicochemical Properties of Deep-Fried Maize-Based Snacks

The maize finger all have bulk densities in a similar range from 0.846 g/mL in spiced 100% maize finger to 0.863 g/mL in spiced 20% soy-maize finger, while the chin-chin samples had almost the same bulk density with a percentage difference of 0.2 g/mL (Table 3). There was no significant (*p* > 0.05) difference between the bulk densities of the products. The bulk density is a vital parameter that defines the ease of packaging and conveyance of particulate foods [9].

Color is a critical quality attribute in food, influencing consumer choice [44]. The color of the maize snacks (L*, a*, and b* values), which depicts the degree of lightness, redness, and yellowness, shows no significant difference at *p* > 0.05 across all products. However, 100% maize finger have the highest degree of lightness and yellowness values (5724, 5620; 3003.5, 3083.5), respectively, while spiced fortified maize finger have the highest degree of redness (Table 3). The observed trend agrees with the report of Sha et al. [45], whose degree of redness of corn snacks supplemented with soy and chickpea flour increased with supplementation and degree of yellowness was found in corn snacks developed without supplementation of soy or chickpea flour. Furthermore, Anton et al. [38] reported a slight color impact when corn starch-based extruded snacks were fortified with navy beans. At the same time, a little red bean flour addition resulted in evident color changes. 

### 3.2. Demographic Information of the Respondents for Maize Products 

Table 4 presents the demographic information and the awareness of the maize chin-chin snacks and their consumption frequency. Significantly few respondents acknowledged knowing about the maize chin-chin snack. Monze District, alongside Serenje District, had an awareness level of 1.94%, recording the lowest awareness level. In comparison, Katete and Mkushi districts had a 2.67% awareness level, the highest level recorded out of a total 9.22% awareness level among the correspondents that claimed to be aware of the products. The highest level of ignorance of the maize chin-chin snack was recorded in Serenje District, where 99% of respondents claimed ignorance of the product. The frequency of consumption of the product was extremely low, just as the level of awareness was equally low, with the lowest being recorded in Monze and Serenje districts.

In contrast, Katete and Mkushi districts recorded a 13.16% daily consumption rate. It was observed that male respondents consumed more of the product, with 59.32% of the total respondents being male. More male respondents consumed the product than females in all the study locations except Serenje District, where awareness is generally low.

There was a very low awareness level of the maize finger’ product, with just 12.9% of total respondents claiming knowledge of the product; 2.3% of which are in Mkushi District, where they had the lowest awareness level. The highest awareness level was found in Serenje District. Maize finger snack is least consumed daily in Katete District and Serenje District, having 1% daily consumption frequency. Some 58.31% of total respondents are female and most of them are from the Katete District, while Monze and Mkushi districts share an almost equal percentage of total female respondents.

### 3.3. Consumer Preference Ratings for Maize Chin-Chin and Maize Finger

Table 5 shows the analysis of variance (ANOVA) results of the effects of gender, product types and, location on the sensory attributes of maize chin-chin and maize finger (Kokoro), respectively. Gender, Product type, and District (Location) had significant effects (*p* < 0.001) on all the sensory attributes for both maize chin-chin and maize finger except appearance and aroma that showed no significant effect of product type and location, respectively. This implies that the sensory attributes ratings were gender-dependent. This follows a similar pattern reported by Alamu et al. [46], who inferred that snack foods’ preference depends mostly on biological factors (genetic), which are gender related. To buttress the observation that sensory attributes are gender-dependent, [47] reported that gender significantly affects tenderness, flavor intensity, and overall acceptability on goose meat. The appearance attribute is not essential in rating maize-based snacks studied, and aroma does not affect the rating of maize snacks across the districts. In a study involving consumer acceptability of two variants of maize baobab snacks, all other sensory parameters such as appearance and aroma were not significant in the overall ratings except taste [48]. Taste and texture are the driving sensory attributes observed for these maize snacks. Jaworska and Hoffmann [49] evaluated the relationship between texture and other sensory attributes on potato chips. It was revealed that texture significantly correlated with overall sensory quality and consumer acceptance.

### 3.4. Consumer Preference Ratings for Maize Chin-Chin Products by District and across Districts

Table 6 shows the consumer preference rating for maize chin-chin by district and across the districts. The appearance, taste, and texture for maize chin-chin are not significantly different at *p* < 0.05 for all locations except Serenje. The appearance ratings of maize chin-chin snacks ranged from 4.8 ± 0.44 for soy-maize chin-chin at Monze District to 4.8 ± 0.51 at Mkushi district for 100% maize chin-chin. It was not significantly different at a value of *p* < 0.05 except in Serenje. The aroma of the various products across the five districts had no significant difference (*p* > 0.05). The aroma is the only attribute that does not significantly differ among the five districts ranging from 4.2 ± 0.82 to 4.7 ± 0.52. The taste attribute was significantly different at a value of *p* < 0.05, ranging from 4.1 ± 0.99 for 100% maize chin-chin at Serenje District to 4.7 ± 0.6 for soy-maize chin-chin at Mkushi District. The overall acceptability ranged from 4.1 ± 1.01 to 4.8 ± 0.44 for 100% maize chin-chin in Serenje District to soy-maize chin-chin product in Mkushi District, respectively, with no significant difference at a value of *p* < 0.05 except Serenje. Moreover, maize chin-chin’s overall acceptability for Serenje District is significantly different at *p* < 0.05 compared with other districts. In all the districts, the soy-fortified maize chin-chin variant has the higher overall acceptability ratings except in Katete. Many researchers had reported decreased acceptability of maize snacks when legume fortification increased [18,22], while some reported otherwise [16]. The nature of the legume and the processes subjected to before utilization in fortification may determine the level of product acceptability. For instance, a product fortified with roasted or malted soy flour tends to enjoy higher sensory acceptability than another product fortified with unroasted or unmalted soy flour. This is because roasting or malting decreases the inherent in them to the least, improves flavor and color, and denatures protein, thereby improving digestibility [50].

In Monze and Mkushi, fortified maize chin-chin taste has the highest rating of 4.60 ± 0.84 and 4.70 ± 0.60, respectively. While in Katete and Serenje, the most rated attributes are aroma (4.60 ± 0.74) and appearance (4.80 ± 0.48) for the fortified maize chin-chin. The lowest sensory ratings go to Serenje District; this same trend was observed by [25] in evaluating sensory properties for wheat and cassava chin-chin variants.

There is a significant (*p* < 0.05) difference between the maize products for all sensory attributes except appearance. Nevertheless, fortified maize chin-chin has the highest ratings for all sensory attributes. 

Table 7 shows the preferences for maize finger variants according to districts and across the districts. There seems to be no significant difference for sensory parameters in all locations except in Serenje District. All districts had 100% spiced maize finger, the most rated for all sensory parameters, and the next in the rating was the spiced fortified maize finger, which applies to all districts except Mkushi. The maize finger with the least preferred aroma were observed to be the spiced soy-maize finger at the Mkushi District with a value of 3.9 ± 1.21, while the product with the most preferred aroma was spiced 100% maize finger with a value of 4.4 ± 1.00. The spiced soy-maize had the lowest aroma rating due to its characteristic beany flavor [51]. However, the trend observed is that the maize finger product with the lowest overall acceptance level is the 100% maize finger with a mean value of 3.9 ± 0.93, 3.7 ± 1.18, and 3.6 ± 0.89, at Monze, Katete, and Serenje districts, respectively. This strongly suggests that the inclusion of soy flour positively influenced the maize finger’ taste. Uzor-Peters et al. [51] reported a similar trend when they fortified maize finger with defatted soya cake flour in different ratios of 1:1, 7:3, and 9:1. The maize-soy finger at a ratio of 9:1 had the highest rating for all sensory attributes.

The most rated maize finger variant is the spiced 100% maize finger, while the next in the rating is spiced soy-maize finger. The spice used on these products is onions (*Allium cepa* L.), a vital vegetable crop used as a spice and food ingredient due to its scent, taste, and intense flavor [52]. It has been reported to be effective against cardiovascular disease, hypolipidemic, anti-hypertensive, anti-diabetic, antithrombotic, and anti-hyperhomocysteinemia effects, and many other biological activities such as antimicrobial, antioxidant, anticarcinogenic, antimutagenic, antiasthmatic, immunomodulatory, and prebiotic activities. 

In contrast, the onion’s strong flavor successfully masked the beany flavor associated with soy flour used to fortify the spiced soy-maize finger. Thus, the spice’s inclusion serves a dual purpose; improvement of sensory attributes and health-promoting activities.

## 4. Conclusions

Maize snacks have proven to contain high nutritional content whose quality can be further improved by fortification with soybean flour with a reasonable level of acceptability. This will help to create a diversity of nutrient-dense foods, thereby shrinking the pool of an under-nourished population. Maize chin-chin fortified with 20% soy flour has the highest acceptability in Monze, Katete, and Mkushi districts. In comparison, the spiced 100% maize finger enjoyed the highest acceptability across all districts except in Mkushi, where the spiced soy-maize finger had the least rating for aroma. This indicates that the soy flour used for fortification may undergo more processing operations or an entirely new processing method to reduce the beany flavor of soybean. Furthermore, two or more legumes may be used to fortify more maize products as protein digestibility and availability will be researched. However, it is essential to know that the Zambians’ nutritional status will be considerably upgraded.

Thus, maize in nutritious, healthy snacks in Zambia will benefit the maize value chain’s improvement by placing a higher demand on the produce, thereby increasing its economic value and providing job opportunities. There is an urgent need to train farmers and processors to commercialize these relatively new products. 

## Figures and Tables

**Table 1:** Recipe for maize-based snacks.

Ingredients/Quantity		Products			
	Plain maize finger	Spiced maize finger	Maize-soy finger	Maize chin-chin	Maize-soy chin-chin
Maize flour	500 g	500 g	500 g	500 g	500 g
Soy flour	–	–	125 g	–	125 g
Salt	5 g	5 g	5 g	–	–
Water	625 mL	620 mL	625 mL	250 mL	500 mL
Sugar	–	–	–	100 g	100 g
Baking powder	–-	–	–	10 g	10 g
Margarine	–	–	–	40 g	40 g
Eggs	–	–	–	2 medium-sized	2 medium-sized
Onions	–	2 medium-sized	–	–	–

**Table 2:** Nutritional and Antinutritional properties of deep-fried maize-based snacks.

Parameters														
Products	MC (%)	Ash (%)	Fat (%)	Protein (%)	Amylose (%)	Amylopectin (%)	Sugar (%)	Starch (%)	TCHO (%)	TDCHO (%)	TNDCHO (%)	Energy Value (Kcal/100 g)	Tannin (mg/g)	Phytate (%)
100% maize kokoro	2.191 ^a^	1.611 ^c^	15.555 ^b^	9.750 ^ab^	20.494 ^c^	79.506 ^b^	1.881 ^c^	62.730 ^a^	70.893 ^b^	64.611 ^a^	6.282 ^e^	462.565 ^b^	1.130 ^c^	1.454 ^d^
Spiced 100% maize kokoro	2.443 ^a^	1.653 ^c^	12.852 ^c^	9.650 ^b^	24.259 ^b^	75.741 ^c^	2.695 ^b^	51.952 ^c^	73.402 ^a^	54.648 ^c^	18.755 ^b^	447.874 ^c^	1.410 ^b^	1.644 ^c^
Spiced 20% soy-maize kokoro	2.342 ^a^	2.453 ^a^	15.431 ^b^	10.656 ^a^	25.475 ^a^	74.525 ^d^	3.411 ^a^	48.712 ^d^	69.117 ^c^	52.124 ^d^	16.994 ^c^	457.976 ^b^	1.552 ^b^	2.332 ^a^
100% maize chin-chin	1.332 ^b^	2.340 ^b^	18.619 ^a^	8.605 ^c^	20.114 ^d^	79.886 ^a^	3.450 ^a^	56.126 ^b^	69.104 ^c^	59.576 ^b^	9.528 ^d^	478.411 ^a^	1.538 ^b^	1.236 ^e^
20% Soy-maize chin-chin	1.346 ^b^	2.455 ^d^	18.322 ^a^	9.188 ^bc^	20.722 ^c^	79.278 ^b^	3.393 ^a^	42.137 ^e^	69.689 ^c^	45.530 ^e^	24.158 ^a^	480.407 ^a^	1.975 ^a^	1.869 ^b^
Minimum	1.298	1.447	12.631	8.560	20.076	74.449	1.843	42.137	69.034	45.511	6.066	446.137	1.111	1.234
Maximum	2.598	2.475	18.668	10.938	25.551	79.924	3.488	62.810	73.654	64.653	24.284	480.816	2.050	2.362
Mean	1.931	1.902	16.156	9.570	22.213	77.787	2.966	52.332	70.441	55.298	15.143	465.447	1.521	1.707
Std. deviation	0.529	0.433	2.244	0.735	2.330	2.330	0.645	7.299	1.715	6.847	6.800	13.074	0.291	0.397
Pr > F (Products)	**	***	***	**	***	***	***	***	***	***	***	***	***	***

^a^ Parameters were analyzed in duplicate. Mean values in the same column with different letters are significantly different at *p* < 0.05. **, significant at *p* < 0.01; ***, significant at *p* < 0.001. MC = Moisture content; TCHO = Total carbohydrate; TDCHO = Total digestible carbohydrate; TNDCHO = Total non-digestible carbohydrate.

**Table 3:** Physicochemical properties of deep-fried maize-based snack.

Products	pH	Bulk Density (g/mL)	L*	a*	b*
100% maize finger	6.740 ^c^	0.853 ^a^	5620 ^a^	760.0 ^a^	3003.5 ^a^
spiced 100% maize finger	6.790 ^c^	0.846 ^a^	5724 ^a^	638.5 ^a^	3083.5 ^a^
Spiced 20% soy-maize finger	6.515 ^d^	0.863 ^a^	5106 ^a^	854.5 ^a^	2803.5 ^a^
100% maize chin-chin	7.130 ^b^	0.780 ^a^	5361 ^a^	732.5 ^a^	2796.5 ^a^
20% soy-maize chin-chin	7.430 ^a^	0.782 ^a^	5374 ^a^	719.0 ^a^	2720.5 ^a^
Minimum	6.510	0.760	4991	389	2693
Maximum	7.440	0.939	5814	907	3339
Mean	6.921	0.825	5437	741	2882
Std. deviation	0.340	0.054	240	141	197
Pr > F (Products)	***	Ns	**	Ns	Ns

^a^ Parameters were analyzed in duplicate. Mean values in the same column with different letters are significantly different at *p* < 0.05. ns, not significant at *p* < 0.05; **, significant at *p* < 0.01; ***, significant at *p* < 0.001.

**Table 4:** Demographic information of the respondents for maize-based products.

**Maize Chin-Chin**		**Monze**	**Katete**	**Serenje**	**Mkushi**
Variables		N (%)	N (%)	N (%)	N (%)
Gender	Female	42 (10.17)	30 (7.26)	61 (14.77)	35 (8.47)
	Male	62 (15.01)	79 (19.13)	47 (11.38)	57 (13.8)
Age (year)	Mean ± SD	35.1 ± 13.27	38.3 ± 12.33	43.8 ± 12.26	41.0 ± 11.42
	Minimum	14	18	21	20
	Maximum	72	105	76	73
Awareness of maize chin-chin N(%) Yes No	Female Male	8(1.94) 96(23.3)	11.6(2.67) 98(23.73)	8(1.94) 99(24.03)	11(2.67) 81(19.66)
**Maize Kokoro**		**Monze**	**Katete**	**Serenje**	**Mkushi**
Variables		N (%)	N (%)	N (%)	N (%)
Gender	Female	59 (14.54)	77 (19.11)	40 (9.93)	59 (14.64)
	Male	43 (10.67)	29 (7.2)	65 (16.13)	31 (7.69)
Age (year)	Mean ± SD	35.6 ± 14.27	38.3 ± 11.24	42.9 ± 12.91	40.7 ± 12.1
	Minimum	14	21	18	15
	Maximum	72	84	79	73
Gender	Female Male	59(14.54) 43(10.67)	77(19.11) 29(7.2)	40(9.93) 65(16.13)	59(14.64) 31(7.69)

**Table 5:** ANOVA of consumer preference ratings of maize-based products.

**Maize Chin-Chin**	**Product Attributes**					
Source	DF	Appearance	Aroma	Taste	Texture	Overall acceptability
Gender	1	2.2196 **	5.3176 ***	8.1343 ***	6.7947 ***	7.7645 ***
Product	1	1.1634	17.4334 ***	22.7228 ***	17.1441 ***	15.1864 ***
District	3	2.5717 **	1.27899	3.27146 **	3.3450 **	5.2905 ***
Error	821	0.5644	0.6329	0.7062	0.8538	0.5983
**Maize Kokoro**	**Product Attributes**					
Source	DF	Appearance	Aroma	Taste	Texture	Overall acceptability
Gender	1	5.3334 **	5.5007 ***	19.2687 ***	24.3147 ***	17.0796 ***
Product	2	8.8309	11.9139 ***	47.2613 ***	59.0050 ***	37.2600 ***
District	3	3.0000 **	1.2250	0.6070 **	1.1879 **	3.6325 ***
Error	1202	0.8500	1.0534	2.4767	1.2176	0.9664

**, significant at *p* < 0.01; ***, significant at *p* < 0.001.

**Table 6:** Consumer preference ratings for maize chin-chin products by district and across the districts.

**Attributes**		**Appearance**			**Aroma**		**Taste**		**Texture**		**OA**	
**District**	**Sample**	**N**	**Mean ± SD**	**CV**	**Mean ± SD**	**CV**	**Mean ± SD**	**CV**	**Mean ± SD**	**CV**	**Mean ± SD**	**CV**
**Monze**	Soy-maize chin-chin	104	4.80 ± 0.44 ^a^	9.2	4.60 ± 0.64 ^a^	13.7	4.60 ± 0.84 ^a^	18.3	4.50 ± 0.87 ^a^	19.42	4.60 ± 0.72 ^a^	15.55
	100% maize chin-chin	104	4.80 ± 0.45 ^a^	21.5	4.20 ± 0.92 ^a^	21.9	4.40 ± 0.79 ^a^	18.1	4.10 ± 1.08 ^a^	26.29	4.30 ± 0.83 ^a^	19.42
**Katete**	Soy-maize chin-chin	109	4.80 ± 0.46 ^a^	15.5	4.60 ± 0.74 ^a^	16.2	4.60 ± 0.72 ^a^	15.6	4.50 ± 0.75 ^a^	16.72	4.60 ± 0.63 ^a^	13.6
	100% maize chin-chin	109	4.80 ± 0.47 ^a^	15.8	4.30 ± 0.93 ^a^	21.6	4.40 ± 0.89 ^a^	20.4	4.30 ± 0.98 ^a^	22.7	4.50 ± 0.76 ^a^	17.14
**Serenje**	Soy-maize chin-chin	108	4.80 ± 0.48 ^b^	19.3	4.30 ± 0.76 ^a^	17.5	4.40 ± 0.75 ^b^	17	4.10 ± 0.94 ^b^	22.55	4.30 ± 0.84 ^b^	19.62
	100% maize chin-chin	108	4.80 ± 0.49 ^b^	17.2	4.20 ± 0.82 ^a^	19.3	4.10 ± 0.99 ^b^	24.4	4.10 ± 1 ^b^	24.59	4.10 ± 1.01 ^b^	24.4
**Mkushi**	Soy-maize chin-chin	92	4.80 ± 0.50 ^a^	15.7	4.70 ± 0.52 ^a^	11.1	4.70 ± 0.6 ^a^	12.8	4.60 ± 0.68 ^a^	14.73	4.80 ± 0.44 ^a^	9.21
	100% maize chin-chin	92	4.80 ± 0.51 ^a^	15.8	4.20 ± 0.9 ^a^	21.6	4.10 ±1.04 ^a^	25.3	4.10 ± 0.99 ^a^	24.36	4.30 ± 0.77 ^a^	18.04
**Ratings across the districts** **Attributes**		**Appearance**			**Aroma**		**Taste**		**Texture**		**OA**	
	**Sample**	**N**	**Mean ± SD**	**CV**	**Mean ± SD**	**CV**	**Mean ± SD**	**CV**	**Mean ± SD**	**CV**	**Mean ± SD**	**CV**
	Soy-maize chin chin	413	4.6 ± 0.72 ^a^	15.8	4.5 ± 0.69 ^a^	15.2	4.6 ± 0.74 ^a^	16.3	4.4 ± 0.83 ^a^	18.85	4.6 ± 0.7 ^a^	15.4
	100% maize chin chin	413	4.5 ± 0.79 ^a^	17.7	4.2 ± 0.89 ^b^	21	4.2 ± 0.94 ^b^	22.1	4.1 ± 1.01 ^b^	24.5	4.3 ± 0.86 ^b^	19.99
	Total	826	4.5 ± 0.76	16.8	4.4 ± 0.81	18.4	4.4 ± 0.86	19.6	4.3 ± 0.94	21.93	4.4 ± 0.8	17.97

Mean values in the same column with different letters are significantly different at *p* < 0.05; OA = overall acceptability.

**Table 7:** Consumer preference ratings for maize finger products by district and across the districts.

**Attributes**		**Appearance**			**Aroma**		**Taste**		**Texture**		**OA**	
**District**	**Sample**	**N**	**Mean ± SD**	**CV**	**Mean ± SD**	**CV**	**Mean ± SD**	**CV**	**Mean ± SD**	**CV**	**Mean ± SD**	**CV**
**Monze**	100% maize finger	102	4.4 ± 0.9 ^a^	20.21	4.0 ± 0.97 ^a^	23.92	3.7 ± 1.11 ^a^	29.75	3.5 ± 1.04 ^a^	30.06	3.9 ± 0.93 ^a^	23.64
	Spiced 100% maize finger	102	4.5 ± 0.92 ^a^	20.3	4.3 ± 1.03 ^a^	23.98	4.4 ± 0.9 ^a^	20.57	4.2 ± 0.99 ^a^	23.7	4.5 ± 0.75 ^a^	16.83
	Spiced soy-maize finger	102	4.3 ± 0.9 ^a^	20.88	4.0 ± 1.07 ^a^	26.92	3.9 ± 1.16 ^a^	29.98	3.9 ± 1.17 ^a^	29.74	4.0 ± 1.05 ^a^	26.33
**Katete**	100% maize finger	106	4.3 ± 0.93 ^a^	21.56	3.9 ± 1.27 ^a^	32.21	3.6 ± 1.26 ^a^	34.93	3.4 ± 1.35 ^a^	39.92	3.7 ± 1.18 ^a^	31.63
	Spiced 100% maize finger	106	4.6 ± 0.81 ^a^	17.46	4.5 ± 0.94 ^a^	20.86	4.4 ± 1 ^a^	22.88	4.3 ± 1.08 ^a^	25.11	4.5 ± 0.94 ^a^	20.72
	Spiced soy-maize finger	106	4.4 ± 0.87 ^a^	19.79	4.3 ± 1.01^a^	23.37	4.2 ± 1.04 ^a^	24.72	4.3 ± 1.03 ^a^	23.82	4.4 ± 0.96 ^a^	21.97
**Serenje**	100% maize finger	105	4.1 ± 0.99 ^b^	23.94	4.0 ± 0.93 ^a^	23.26	3.5 ± 1.19 ^a^	33.97	3.4 ± 1.19 ^a^	35.44	3.6 ± 0.89 ^b^	24.34
	Spiced 100% maize finger	105	4.4 ± 0.79 b	17.97	4.3 ± 0.86 ^a^	20.12	4.3 ± 0.92 ^a^	21.4	4.3 ± 0.79 ^a^	18.51	4.4 ± 0.78 ^b^	17.72
	Spiced soy-maize finger	105	4.2 ± 1.02 b	24.51	4.1 ± 0.97 ^a^	23.53	4.2 ± 3.99 ^a^	96.09	4 ± 1.02 ^a^	25.7	3.9 ± 0.98 ^b^	25.35
**Mkushi**	100% maize finger	90	4.4 ± 0.93 a	21.22	4.3 ± 0.95 ^a^	22.17	3.9 ± 1.16 ^a^	30.08	3.8 ± 1.08 ^a^	28.87	4.2 ± 0.93 ^a^	22.25
	Spiced 100% maize finger	90	4.6 ± 0.88 a	19.07	4.4 ± 1.00 ^a^	22.77	4.4 ± 0.99 ^a^	22.63	4.1 ± 1.2 ^a^	29.46	4.4 ± 1.09 ^a^	24.67
	Spiced soy-maize finger	89	4.2 ± 1.12 a	26.4	3.9 ± 1.21 ^a^	30.87	4 ± 1.25 ^a^	31.16	3.9 ± 1.16 ^a^	29.66	4.0 ± 1.17 ^a^	29.27
**Ratings across the districts** **Attributes**		**Appearance**			**Aroma**		**Taste**		**Texture**		**OA**	
	Sample	N	Mean ± SD	CV	Mean ± SD	CV	Mean ± SD	CV	Mean ± SD	CV	Mean ± SD	CV
	100% maize finger	403	4.3 ± 0.94 ^b^	21.84	4.1 ± 1.04 ^b^	25.77	3.7 ± 1.19 ^c^	32.33	3.5 ± 1.18 c	33.98	3.9 ± 1.01 ^c^	26.1
	Spiced 100% maize finger	403	4.5 ± 0.85 ^a^	18.73	4.4 ± 0.96 ^a^	21.95	4.4 ± 0.95 ^a^	21.8	4.2 ± 1.02 ^a^	24.19	4.5 ± 0.89 ^a^	20.01
	Spiced soy-maize finger	402	4.3 ± 0.98 ^b^	22.84	4.1 ± 1.07 ^b^	26.15	4.1 ± 2.26 ^b^	55.59	4 ± 1.1 ^b^	27.29	4.1 ± 1.05 ^b^	25.9
	Total	1208	4.4 ± 0.93	21.28	4.2 ± 1.04	24.8	4 ± 1.6	39.59	11.7 ± 3.3	85.46	12.5 ± 2.95	72.01

Mean values in the same column with different letters are significantly different at *p* < 0.05. OA = overall acceptability.
